# Clinical Evaluation of Percutaneous Vertebroplasty in a Patient with Paraplegia and Immobilization Syndrome: A Case Report

**DOI:** 10.1155/2013/356109

**Published:** 2013-03-14

**Authors:** Salvatore Masala, Eros Calabria, Marco Nezzo, Dominique De Vivo, Luca Neroni, Giovanni Simonetti

**Affiliations:** Department of Diagnostic and Molecular Imaging, Interventional Radiology and Radiation Therapy, Fondazione Policlinico Tor Vergata, Viale Oxford 81, 00133 Rome, Italy

## Abstract

We will discuss a potential role of percutaneous vertebroplasty (PVP) in the management of a patient with immobilization syndrome due to paraplegia and vertebral osteoporotic fractures. While PVP is commonly used for the treatment of osteoporotic thoracolumbar vertebral compression fractures, its role in vertebral stabilization in patient with immobilization syndrome has not been reported in the literature. A 73-year-old woman affected by immobilization syndrome due to paraplegia and vertebral osteoporotic fractures was treated with PVP of vertebrae D12, L1, and L4. After PVP, the patient did not need any antalgic therapy, and there was a significant improvement regarding mobilization, performance of physiological functions, daily management of personal care, and treatment of decubitus ulcers, increasing life quality and psychological well-being.

## 1. Introduction 

Immobilization syndrome is a complex multisystem disorder characterized by reduced or absent autonomy movement that leads to a negative impact on the psychophysical state for the affected patients. 

Immobility promotes the development of osteoporosis, disease of high social importance, that represents a serious public health emergency in all the countries where the demographic trend leans towards a progressive increase in life expectancy [1]. 

Patients with spinal cord injury (SCI) are often associated with severe osteoporosis and risk of bone fracture.

Osteoporotic fractures are considered atraumatic because of being generated by minor injuries; the vertebral bodies, the distal radius, and the femoral neck are the most likely locations. 

The severity of vertebral fractures correlates with the intensity of the back pain and the consequent functional impairment, leading to further movement limitation for the patient. 

The failure of medical therapy is defined as a mild or no pain relief with analgesic therapy after a period of treatment of at least four to six weeks [2–4] or side effects attributable to drugs (excessive sedation, confusion, and constipation). 

The pain also induces depression, promotes a negative psychological attitude toward oneself and the environment, and reduces the motivation to participate in family and social life, with negative impact on quality of life. 

Percutaneous vertebroplasty (PVP) is a minimally invasive technique employing the injection of liquid polymethylmethacrylate (PMMA) cement into a fractured vertebral body to relieve pain, reinforce the bone, and prevent further vertebral compression [5–7]. 

## 2. Case Report 

In June 2012, a 71-year-old man has been admitted to our Emergency Department with low back pain, dyspnea, and fever. 

The patient entered menopause at the age of 54, and osteoporosis was diagnosed in 2006 using DXA-scan (lumbar spine BMD 0.690 g/cm^2^, *T*-score 2.6 DS, femoral neck BMD 0.547 g/cm^2^, *T*-score 2.5 DS). 

The patient referred posttraumatic subarachnoid haemorrhage in 2007 that resulted in right upper limb plegia and paraplegia and immobilization syndrome complicated by a sacral decubitus ulcer (stage IV). 

Approximately 20 hours after arrival, the patient was transferred to the Department of Internal Medicine. 

During the hospitalization, the analgesic therapy was administered without achieving a complete resolution of the clinical picture, with persistence of low back pain at rest, exacerbated by the mobilization, limiting the performance of the physiological functions and the personal care. 

A radiograph of the lumbar spine revealed morphological and structural alteration of D12 body, right-convex scoliosis of the thoracic spine, and marked reduction in calcium tone. 

**Figure 1:**
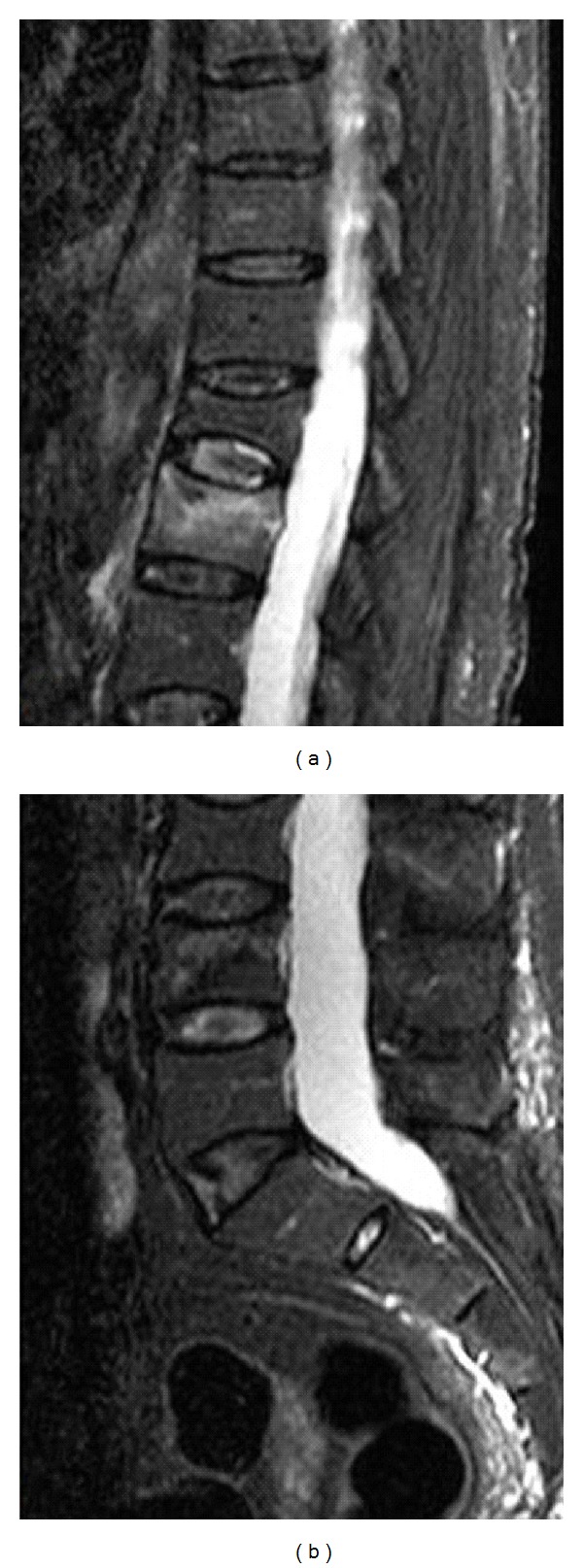
Sagittal STIR MR dorsal (a) and lumbar (b) image. MR image shows deformity of T12 vertebral body, which is chronic; there is no bone marrow edema. The recent fractures of L1 and L4 are much less deformed but have high signal from bone marrow edema.

Magnetic resonance (MR) of the lumbar spine with administration of intravenous gadolinium showed recent vertebral collapses at the level of L1 and L4 bodies and confirmed previous fractures in D12 body (Figure 1). 

After the evaluation of the case by the interventional radiologist, the PVP of vertebrae D12, L1, and L4 was performed. 

PVP is not routinely performed in immobilized patients; in fact, while it is commonly used for the treatment of osteoporotic thoracolumbar vertebral compression fractures, its role in vertebral stabilization in patient with immobilization syndrome has not been reported in the literature [8]. 

Anyway, we decided to proceed to improve quality life of the patient treating the intense pain. 

The preoperative and postoperative pain perceptions were compared using (Visual Analogue Scale) VAS. The latter is nothing but a simple graphical representation of the pain, recorded on a 10 cm numerical scale ranging from zero (no pain) to 10 (maximum pain ever felt). This method allows the patient to bypass the cognitive level of his brain and give a truer representation of pain. 

For assessing the quality of life questionnaire, (Psychological General Well Being Index) PGWBI was administered [9]. 

The patient was informed on the technique, the benefits, and the potential complications of the intervention as well as postprocedure care, and a written informed consent was obtained. 

The hematological examinations including blood coagulation were obtained, and all the values were within the normal range. 

The segment to be treated was identified under fluoroscopic guidance (Allura, Phillips, The Netherlands) on the angiography table with the patient in prone position. 

Local anesthesia consisting of lidocaine 2% (10 mL) and naropine 7.5% (10 mL) and drugs for sedation in spontaneous breathing were administered, and a 13 G needle with left transpedicular approach at the level of D12, L1, and L4 bodies was introduced. 

**Figure 2:**
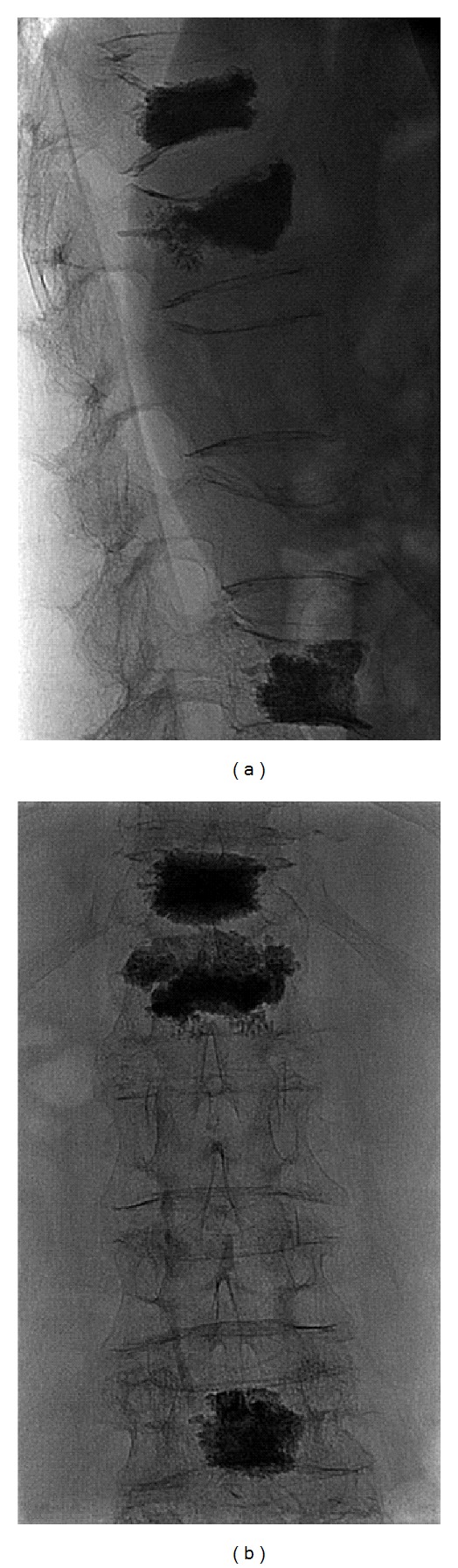
Fluoroscopic biplanar control postprocedure in the lateral (a) and anteroposterior (b). Optimal and uniform distribution of polymethylmethacrylate (PMMA) within the bodies of D12, L1, and L4. The postprocedural fluoroscopic control is necessary to ensure that the PMMA does not extend into the prevertebral space, the dural sac, or in the paravertebral vessels.

About 3 mL of polymethylmethacrylate (PMMA) were injected in every vertebral body with uniform distribution of cementing material without intra- and postprocedural complications (Figure 2). 

After a few hours from the end of the procedure, pain relief was significant. 

Duration of the procedure was about 25 minutes. 

The efficacy of a PVP was demonstrated by a decrease from 9/10 to 4/10 on static VAS score. 

For the evaluation of psychophysical well-being, PGWBI [9] questionnaire was used, including 22 items, each of which presents a series of six possible answers, whose score is proportional to the positivity and well-being over the last four weeks. 

The scale consists of six domains or dimensions: anxiety, depression, positivity and well-being, self-control, general health, and vitality. 

During hospitalization, the patient obtained a score of 54, equal to serious illness. At one month after PVP, the patient has scored 65, equivalent to moderate discomfort. 

After the procedure, the patient did not need any antalgic therapy, and there was a significant improvement on passive mobilization, performance of physiological functions, daily management of personal care, and maintaining skin integrity for the treatment of decubitus ulcers, with a significant increase of life quality and psychological well-being. 

## 3. Discussion 

We reported a case of PVP performed in a patient with paraplegia and immobilization syndrome to improve the life quality and psychological well-being. 

In medical experience, pain is one of the most important manifestations of the disease, and it is among the symptoms that tend to further undermine the life quality, affects abilities to perform daily functions, and negatively affects the emotional sphere, especially in older people with polypathologies which determine clinical and management vulnerability, which adds up to their biological fragility. 

In our paper, the patient, immobilized for about five years due to paraplegia and plegia of right upper limb, presents pain in the lumbar mobilization, in carrying out biological functions, in the daily management of personal hygiene, and treatment of decubitus ulcer, with negative impact on psychophysical well-being. 

For these reasons, given the failure of conservative treatment, it was decided to submit the patient to the PVP at the level of D12, L1, and L4 bodies, obtaining a rapid regression of pain and improving life quality of patient and family, pivotal in helping the not self-sufficient patient. 

The main limitation of this study is the small size of the cohort of patients and the short-term followup, but the results that we obtained in our preliminary experience are encouraging, even if they need a confirmation by further studies involving a larger cohort. In conclusion, taking into account the minimally invasive nature, the short duration, the low cost of the technique, and the improvement of life quality, PVP could be considered a new potential indication in the management of patients with spinal cord injury with associated pathologic vertebral fractures.

## Abbreviations

PVP: Percutaneous vertebroplasty
SCI: Spinal cord injury
PMMA: Polymethylmethacrylate
MR: Magnetic resonance
VAS: Visual analogue scale
PGWBI: Psychological general well being index.
